# Supplementary figures and images for: Genomic characterization of a severe West Nile Virus transmission season using a single reaction amplicon sequencing approach
Source: PLoS Negl Trop Dis. 2026 Jan 21;20(1):e0013931. doi: 10.1371/journal.pntd.0013931 (PMC12844533; doi:10.1371/journal.pntd.0013931)

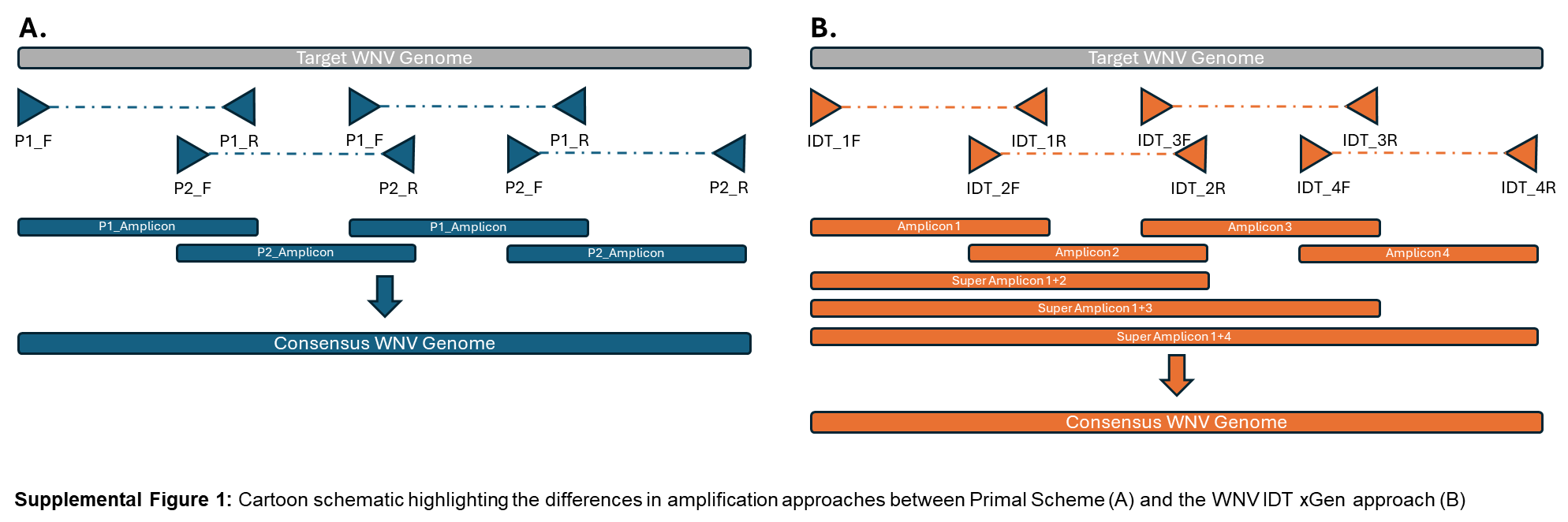

Supplement: S1 Fig — (TIF) [file pntd.0013931.s001.tif]

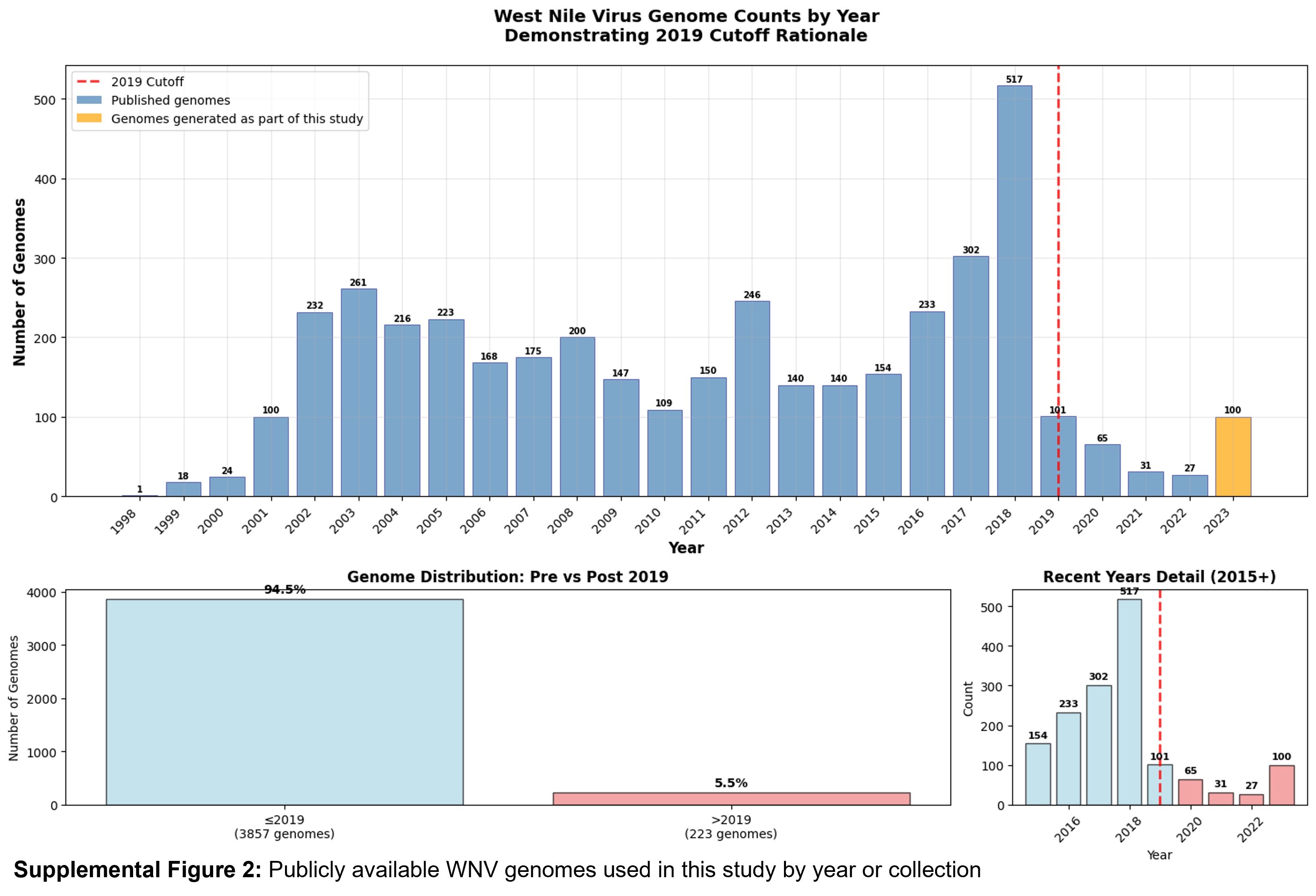

Supplement: S2 Fig — (TIF) [file pntd.0013931.s002.tif]

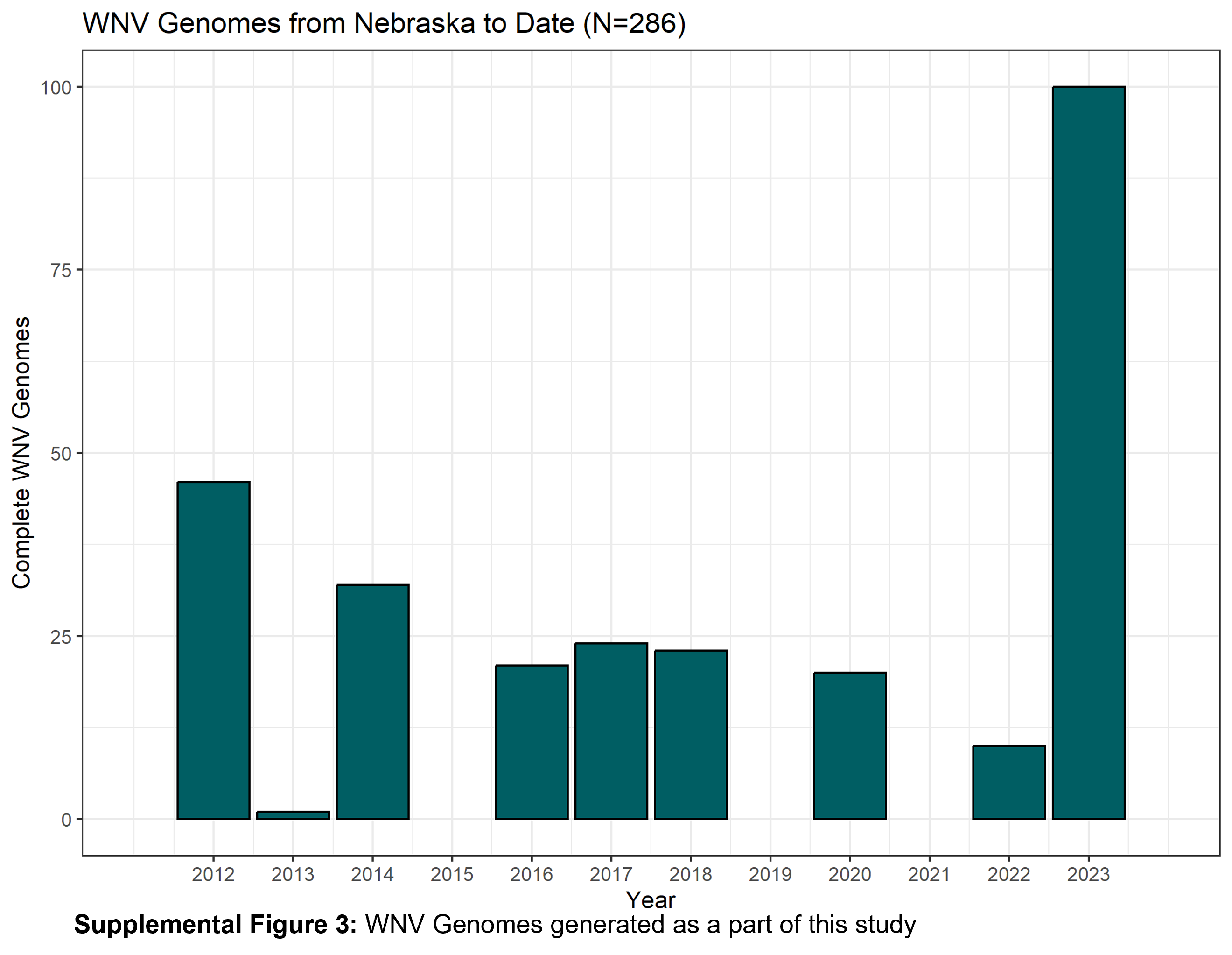

Supplement: S3 Fig — (TIF) [file pntd.0013931.s003.tif]
